# Oxidative stress as an indicator of lower quality eucalyptus for pulp and paper industry

**DOI:** 10.1186/1753-6561-5-S7-P156

**Published:** 2011-09-13

**Authors:** Dahyana Britto, Carlos Pirovani, Esteban Gonzalez, João Silva, Abelmon Gesteira, Júlio Cascardo

**Affiliations:** 1 Center of Biothecnology and Genetics, Dep. of Genetics and Molecular Biology, Universidade Estadual de Santa Cruz - UESC, Ilhéus, BA, 45662-900, Brazil; 2 Suzano Paper and Cellulose S.A., Mucuri, BA, 45936-000, Brazil; 3 Brazilian Enterprise for Agricultural Research cassava and fruit - EMBRAPA, Cruz das Almas, BA, 44380-000, Brazil; 4 in memorian

## Background

Wood is the most important natural and renewable source of infinite energy, but this is not their only function, it also has great significance to the buildings and other products [1]. Eucalyptus, the genus that includes more than 500 species, is among the key forest species planted around the world to solid wood, and mainly for the pulp and paper industries. Aiming at the sustainable use of timber, supplying the demand and meeting the requirements of the paper industry, genetic breeding programs looking for, among other agronomic traits of interest, improve the quality of wood, that is, lower lignin and extractive, and increase the density wood and pulp yield. Therefore, it is interesting to identify genetic markers that characterize the major events of xylem differentiation, as well as stress response, because it is key factors to determine some properties of wood, affecting their performance and industrial value. Thus, this study aimed to identify genes specifically involved in oxidative stress response and characterization of genes in determining the quality of the wood.

## Materials and methods

To this end, we constructed a Suppression Subtractive Hybridization 3 – SSH3 using two samples of xylem. These samples were extracted from trees of a population of sib eucalyptus from a commercial plantation Suzano with contrasting characteristics to wood quality, among them, lignin content and total amount of extractive, higher for the clone X4, and basic density and cellulose yield, for the largest clone X1, the latter considered as higher for the production of pulp and paper. After identification and selection of genes of interest was the analysis of the expression for Real Time PCR with the same clones of Eucalyptus used in making the library.

## Results

From the sequencing and bioinformatics analysis of the library has been possible to identify a total of 449 sequences and 90 contigs in SSH3. Among the most representative unigenes, annotated by the program Blast2GO versão1.2.7 in SSH3 were identified proteins involved in oxidative stress response and heat shock protein, ABC transporter and glutamine synthetase. After analyzing for real-time PCR for validation of these sequences more representative, was unable to confirm the decreased expression of all genes in clone X1 compared with the X4 clone.

## Conclusions

The result was expected for these clones, since the plants when in oxidative stress caused by abiotic and biotic agents, activates the defense mechanism through the expression of genes involved in stress response, as well as accumulated extractives as polyphenols, tannins and resins [2]. So, the ABC transporter, for example, between genes possibly involved in this process,carries lignin monomers to enhance the cell wall making it difficult the entry of pathogens [3,4]. The small heat shock proteins, also known as molecular chaperones, although not expressed constitutively, are abundant in plants, especially when under stress, as observed by Harndahl and collaborators, in transgenic Arabdopsis thaliana [5]. As glutamine synthetase, this protein is involved in improving the assimilation of nitrogen by the plant [6]. Other words, in a soil poor in nitrogen, such as soil Formation Barriers/Spodosol where these clones were grown, these proteins must play important role in plant growth,once the availability of this nutrient is limiting factor growth of eucalypts [7]. In addition, this protein has an important role in maintaining the health of the plant toattack by pathogens [8]. Considering this, it was note worthy that the signaling proteins were oxidative stress in Eucalyptus clone, and that the result reinforces the idea that proteins involved in oxidative stress response is related to the high concentration of extractive and lignin.

Preliminary tests in Real Time PCR with the juvenile offspring of these clones are already underway. But in order to reaffirm the presence of these genes involved in oxidative stress to the condition of poor quality for the production of pulp and paper, the expression analysis performed in the progenies of appropriate age and assessment of components involved in wood quality are needed.
